# Understanding the multiferroicity in TmMn_2_O_5_ by a magnetically induced ferrielectric model

**DOI:** 10.1038/srep34767

**Published:** 2016-10-07

**Authors:** L. Yang, X. Li, M. F. Liu, P. L. Li, Z. B. Yan, M. Zeng, M. H. Qin, X. S. Gao, J.-M. Liu

**Affiliations:** 1 Institute for Advanced Materials and Laboratory of Quantum Engineering and Quantum Materials, South China Normal University, Guangzhou 510006, China; 2 Laboratory of Solid State Microstructures, Nanjing University, Nanjing 210093, China; 3 Institute for Advanced Materials, Hubei Normal University, Huangshi 435003, China

## Abstract

The magnetically induced electric polarization behaviors in multiferroic TmMn_2_O_5_ in response to varying temperature and magnetic field are carefully investigated by means of a series of characterizations including the high precision pyroelectric current technique. Here polycrystalline rather than single crystal samples are used for avoiding the strong electrically self-polarized effect in single crystals, and various parallel experiments on excluding the thermally excited current contributions are performed. The temperature-dependent electric polarization flop as a major character is identified for different measuring paths. The magneto-current measurements indicate that the electric polarization in the low temperature magnetic phase region has different origin from that in the high temperature magnetic phase. It is suggested that the electric polarization does have multiple components which align along different orientations, including the Mn^3+^-Mn^4+^-Mn^3+^ exchange striction induced polarization *P*_*MM*_, the Tm^3+^-Mn^4+^-Tm^3+^ exchange striction induced polarization *P*_*TM*_, and the low temperature polarization *P*_*LT*_ probably associated with the Tm^3+^ commensurate phase. The observed electric polarization flop can be reasonably explained by the ferrielectric model proposed earlier for DyMn_2_O_5_, where *P*_*MM*_ and *P*_*TM*_ are the two antiparallel components both along the *b*-axis and *P*_*LT*_ may align along the *a*-axis. Finally, several issues on the unusual temperature dependence of ferroelectric polarizations are discussed.

The discovery of magnetically induced ferroelectric (FE) polarization in a number of transition metal oxides which usually have the strong electronic correlation characters represents a milestone for multiferroic physics and materials sciences^1^,^2^,^3^,^4^,^5^,^6^. Taking perovskite ABO_3_ manganites (RMnO_3_) as the most extensively investigated multiferroic systems where R is Y or rare-earth^7^,^8^, two major microscopic mechanisms for magnetically induced ferroelectricity have been well accepted. The first mechanism is the collective ionic displacement generated by the Dzyaloshinskii-Moriya (DM) interaction (asymmetric exchange striction) associated with the spin-orbit coupling in non-collinear spin systems such as orthorhombic RMnO_3_ with R = Ga, Tb, Dy, and so on^3^,^9^,^10^,^11^. The second one is the collective ionic displacement generated by the spin-lattice (symmetric exchange striction) in specific collinear spin systems (e.g. ↑↑↓↓ spin order) such as orthorhombic RMnO_3_ with R = Ho, Y, Tm, Lu, and so on^12^,^13^,^14^,^15^. While some other mechanisms have also been proposed for individual systems^16^,^17^, the two mechanisms represent the main pillars for the physics of magnetically induced ferroelectricity in the so-called type-II multiferroics^4^.

Nevertheless, besides RMnO_3_, another class of manganites, RMn_2_O_5_, which are also orthorhombic in structure and identified as the type-II multiferroics, are believed to have different physics of multiferroicity but not yet well-understood^18^,^19^,^20^,^21^,^22^,^23^,^24^,^25^,^26^,^27^. The FE polarizations in most RMn_2_O_5_ are much larger than those in RMnO_3_ and the multiferroic phase transitions are more complicated^23^,^27^,^28^. The possible reason for such big difference is associated with the stronger spin frustration due to the coexistence of Mn^3+^ and Mn^4+^ spins and more seriously distorted lattice structure^29^. These characters may enable more than three, four, or even more magnetic phase transitions within a narrow range of temperature (*T*) below 40 K. We take DyMn_2_O_5_ as an example, which is a representative one of this RMn_2_O_5_ family of complicated magnetic structure evolution^30^. Upon cooling from the paramagnetic (PM) state above *T* = *T*_*N0*_ ~ 43 K, a series of incommensurate (IC) antiferromagnetic (ICM) and commensurate (C) antiferromagnetic (CM) ordering events are identified, where the moments are believed to be from the Mn spins. It is suggested that DyMn_2_O_5_ transits into a non-FE ICM phase below *T*_*N0*_ and then enters a FE CM phase below *T*_*N1*_ ~ 40 K. The magnetic phase transitions at *T*_*N2*_ ~ 28 K mark the gradual transition from the FE CM phase to the non-FE ICM phase until *T*_*Dy*_ ~ 9 K below the Dy^3+^ spins form the independent CM order.

Based on the magnetic scenario highlighted above, the FE behaviors of RMn_2_O_5_ members have been discussed in details^18^,^19^,^20^,^29^,^30^,^31^. The common characters in terms of ferroelectricity, excluding those individual features for respective members, can be summarized from several aspects^11^,^31^. First, an electric polarization *P* (denoted as *P*_*C*_) appears in the CM phase below *T*_*N1*_ and above *T*_*N2*_, which is quite large. The measured *P* drops rapidly down to another *P* (denoted as *P*_*IC*_) once the CM phase is gradually and partially replaced by the ICM phase below *T*_*N2*_. The *P*_*IC*_ can possibly be negative in some cases and positive in others. For reference, the measured *P*(*T*) data below *T*_*N1*_, taken from literature for several compounds using the conventional pyroelectric current technique are schematically drawn in Fig. 1(a)^31^,^32^. Second, the two components *P*_*C*_ and *P*_*IC*_ can be separated by assuming various mechanisms for them. The two cases, taking R = Tm and R = Y, are plotted in Fig. 1(b,c) for a guide of eyes^31^,^33^. It has been suggested that component *P*_*C*_ is related to the symmetric exchange striction, i.e. *P*_*C*_ ~ (*S*_*i*_·*S*_*j*_) with *S*_*i*_ and *S*_*j*_ the spin moments at two neighboring sites *i* and *j*, while component *P*_*IC*_ is ascribed to the asymmetric exchange striction, i.e. *P*_*IC*_ ~ (*S*_*i*_×*S*_*j*_). Both *P*_*C*_ and *P*_*IC*_ align along the *b*-axis. It should be mentioned here that for TmMn_2_O_5_, third electric polarization *P*_*LT*_ along the *a*-axis is believed below *T* ~ *T*_*Tm*_ ~ 5–6 K, which is associated with a long-range commensurate (LCM) ordering of Tm^3+^ spins^34^,^35^. Third, it was revealed that the response of polarization below *T*_*N2*_, i.e. *P*_*IC*_, against magnetic field *H*, is much more remarkable than that of polarization *P*_*C*_ between *T*_*N1*_ and *T*_*N2*_. This effect fits the scenario of *P*_*IC*_ ~ (*S*_*i*_×*S*_*j*_), noting that the noncollinear spin structure is usually more sensitive to varying *H* than the collinear structure^36^,^38^.

Nevertheless, one has several reasons to question the above highlighted scenario on the multiferroicity of RMn_2_O_5_. First, it is seen that the *P*(*T*) dependences of these compounds above *T*_*N2*_, i.e. *P*_*C*_(*T*), are roughly similar for different compounds, but the *P*(*T*) dependences below *T*_*N2*_, i.e. *P*_*IC*_(*T*), are very different from each other with no rational correlation between the *P*_*IC*_(*T*) behavior and the R site ionic size or magnetic moment^22^,^24^. This suggests that the measured *P*_*IC*_ contain some unknown contributions other than *P*_*IC*_ ~ (*S*_*i*_×*S*_*j*_). Second, it is noted that all these data were obtained in single crystal samples grown by flux method. These single crystals are claimed to have strong self-polarized effect which would self-polarize the FE domains^32^,^33^, leading to the strong path-relevance of the measured data. This consequence was often mentioned in literature on RMn_2_O_5_^33^,^35^ to account for those anomalous data on polarization below *T*_*N2*_. In this sense, the proposed relation *P*_*IC*_ ~ (*S*_*i*_×*S*_*j*_) should be questioned too. To avoid this self-polarized effect and its impact on the pyroelectric current data, polycrystalline sample may be even a better choice than single crystal since the small grain size in polycrystalline samples allows a sufficient cancellation of this self-polarized effect if any. Third, the so-called double-wave method (DWM) associated with the Sawyer-Tower circuit was used to measure the polarization-electric field hysteresis loop of these materials^31^. The obtained second hysteresis loops exhibit clear double-loop shape, suggesting possible ferrielectric (FIE) or antiferroelectric (AFE) characters for YMn_2_O_5_ around *T*_*N2*_, reviving the earlier proposed FIE model although a number of unclear issues on this model remains to be identified.

In fact, systematic investigations on the behaviors of *P*(*T*, *H*) in polycrystalline DyMn_2_O_5_ were performed recently using the highly sensitive pyroelectric current technique^29^,^38^,^39^. The main polarization can be illustrated by a FIE model, in which consists of two roughly antiparallel polarization components. This FIE model reasonably explains the main features of the measured multiferroic behaviors of DyMn_2_O_5_ below *T*_*N1*_^29^,^40^. However, this FIE model relies on the R-Mn coupling and it becomes questionable if R is non-magnetic such as R = Y, which remains to be an issue so far. Furthermore and more importantly, additional check of this FIE model with other RMn_2_O_5_ would be necessary for a consideration of generality. In this work, we intend to investigate the multiferroicity of TmMn_2_O_5_. Earlier neutron scattering analysis of the magnetic structure and electric polarization of TmMn_2_O_5_ suggested a qualitatively similar magnetic structure and FE behaviors to those of DyMn_2_O_5_^21^,^34^. The *T*-phase diagram in terms of dielectric permeability and IC/C-AFM phase on single crystal TmMn_2_O_5_ is indeed similar to DyMn_2_O_5_ although the values of *T*_*N0*_, *T*_*N1*_, *T*_*N2*_ are slightly different^34^. This allows us an opportunity to revisit this FIE model for understanding the complicated magnetism-induced ferroelectric behaviors of TmMn_2_O_5_.

## Results

### Multiferroic phase transitions

Before presenting the Multiferroic phase transitions data, we first give a set of microstructural characterizations results. The lattice structure of orthorhombic TmMn_2_O_5_ is schematically shown in Fig. 2(a), which clearly indicates the ordered Mn^3+^ and Mn^4+^ occupation in the lattice. The occupancy of Tm^3+^ ions can be also seen clearly. In Fig. 2(b), the measured and refinement-evaluated data X-ray diffraction (XRD) θ–2θ patterns at room temperature for a sample is presented. Pure orthorhombic structure with space group of Pbam is clearly identified, as confirmed by the high refinement quality factors shown in the inset of Fig. 2(b). The refined lattice parameters are *a* = 0.7216 nm, *b* = 0.8436 nm, and *c* = 0.5654 nm, well consistent with earlier reported results^21^. Furthermore, the scanning electron microscopy (SEM) images of the broken surface of the sample at several scales are shown in Fig. 3(a~c). It is seen that the grains are short-bar like in shape and very dense. The spatial distributions of the species Tm, Mn, and O on an area shown in Fig. 3(d) are presented in Fig. 3(e–o) respectively, revealing the high element homogeneity. The evaluated chemical composition is close to the nominal one within uncertainty of less than 5%.

We discuss the measured specific heat (*C*_*P*_), magnetization (*M*), and dielectric constant (*ε*) as a function of *T* respectively, and the results are summarized in Fig. 4(a~c). In order to exaggerate the features in the *C*_*P*_ ~ *T* curve, the evaluated *d*(*C*_*P*_/*T*)/*dT* ~ *T* curve is inserted in Fig. 4(a) for a comparison. Given the measuring uncertainties from various research groups, the proposed magnetic transition points are nicely reproduced in the present work, with an error of ~ ±1 K, i.e. *T*_*N0*_ ~ 44 K, *T*_*N1*_ ~ 35 K, *T*_*N2*_ ~ 24 K, and *T*_*Tm*_ ~ 6 K^21^,^34^,^35^. As stated earlier^21^,^35^, *T*_*N0*_ marks the transition from the PM phase to the high-temperature ICM phase (probably mixed with small amount of CM phase), and *T*_*N1*_ labels the transition from the ICM phase to the CM phase, followed by the transition from the CM phase to the low temperature ICM phase at *T*_*N2*_. The anomaly at *T*_*Tm*_ indicates the so-called LCM phase associated with the Tm^3+^ spin order and the nature of this LCM ordering remains elusive.

It should be mentioned that the anomaly in the *d*(*C*_*P*_/*T*)/*dT* ~ *T* curve around *T*_*N2*_ is not remarkable, while some other weak anomalies between *T*_*N1*_ and *T*_*Tm*_ have not yet been properly assigned. The weak feature around *T*_*N2*_ seems to suggest that the CM-ICM transition may not be typical and the CM and ICM coexistence in this *T*-range is highly possible. On the other hand, the Tm-Mn coupling should be strong although no specific indication in the *d*(*C*_*P*_/*T*)/*dT* ~ *T* curve can be found. The well-ordered magnetic structure including the R^3+^ spin ordering in the high-*T* range for TmMn_2_O_5_, similar to the cases of DyMn_2_O_5_ and GdMn_2_O_5_ for instance^19^,^27^, confirms the strong Tm-Mn coupling.

Nevertheless, different from the *C*_*P*_ data, the measured *M*(*T*) curve does not show non-trivial feature but a smooth and monotonous increasing with decreasing *T* over the whole *T*-range, as shown in Fig. 4(b). No separation between the ZFC and FC curves and no remarkable anomaly at *T*_*N0*_, *T*_*N1*_, *T*_*N2*_, and even *T*_*Tm*_ can be identified. Obviously the magnetization signals are mainly from Tm^3+^ moment since it is much larger than the Mn^3+^/Mn^4+^ moment. The smooth *M* ~ *T* curve over the whole *T*-range also suggests that the Tm^3+^ spin ordering is already gradually developed far above *T*_*Tm*_ ~ 6 K, most likely induced by the strong Tm-Mn coupling. Otherwise, an anomaly around *T*_*Tm*_ should be observable if the Tm^3+^ spin structure is paramagnetic above *T*_*Tm*_.

The measured *ε*(*T*) data provide additional indication of the multiferroic phase transitions and one set of data at *f* = 1.0 MHz are plotted in Fig. 4(b), while the *ε*(*T*) curves at different *f* show similar features. Besides the relatively sharp peak around *T*_*N1*_ and small peak right around *T*_*Tm*_, a smeared and broad shoulder around *T*_*N2*_ can be found. The sharp peak at ~*T*_*N1*_ certainly marks the FE transition corresponding to the ICM-CM transition. The small peak at ~*T*_*Tm*_ indicates another FE transition which should be related with the LCM ordering at *T*_*Tm*_. The intermediate shoulder (bump) seems to characterize the gradual or diffusive FE transition covering the broad *T*-range around *T*_*N2*_. It was reported that the single-crystal samples do show a weak jump around *T*_*N2*_^34^,^41^, which becomes a shoulder here.

**Figure 1:**
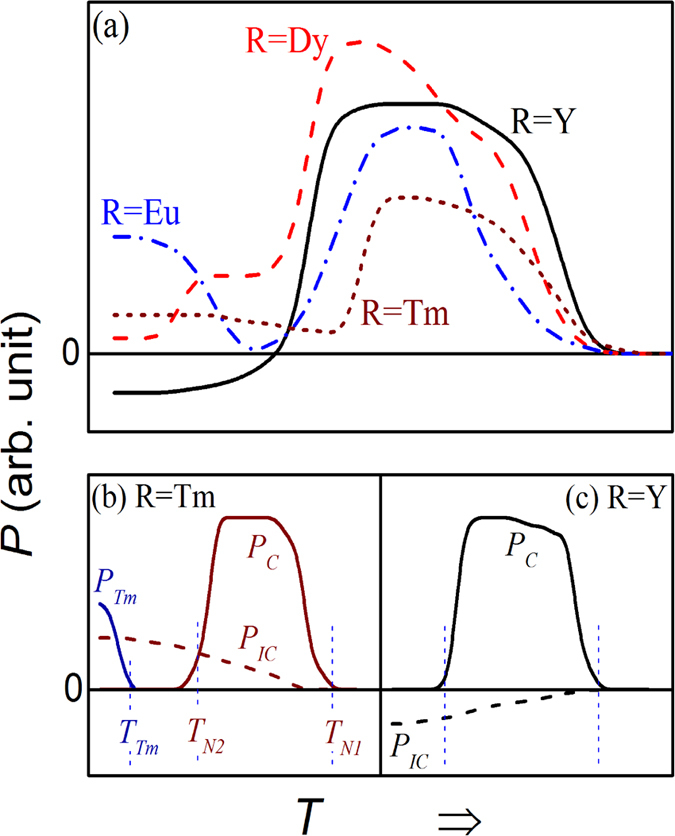
Schematic presentations of ferroelectric polarization *P*(*T*) for several members of RMn_2_O_5_ for a guide of eyes. (**a**) The *P*(*T*) curves for R = Y, Dy, Eu, and Tm. (**b**) The proposed polarization components as a function of *T* respectively for R = Tm, where the claimed origins for *P*_*C*_, *P*_*IC*_, and *P*_*LT*_ are described in text. (**c**) The proposed polarization components as a function of *T* respectively for R = Y. These curves are sketched from the data reported in ref. 31.

**Figure 2:**
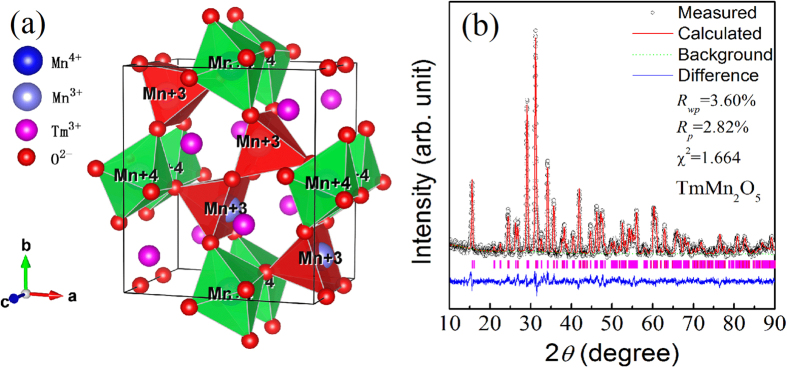
(**a**) A schematic drawing of lattice structure of TmMn_2_O_5_. (**b**) Measured XRD *θ*-2*θ* spectrum of polycrystalline TmMn_2_O_5_ sample and the Rietveld method refined data for comparison. The reliability factors *R*_*wp*_, *R*_*p*_, and χ^2^ of the refinement are labeled.

**Figure 3:**
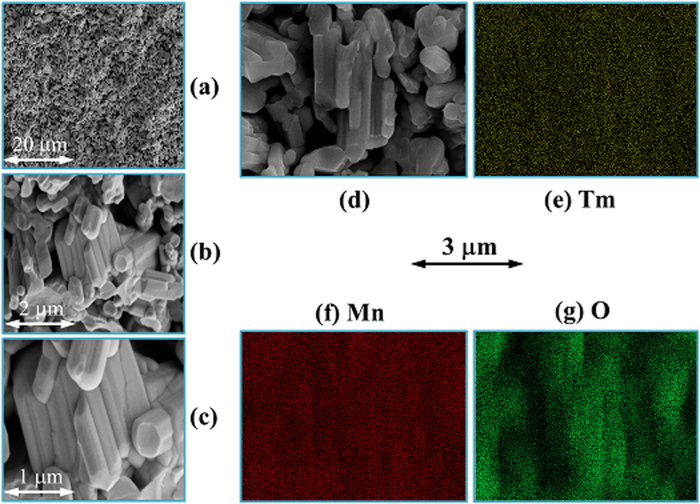
SEM images of the polycrystalline sample (**a–d**). The planar composition distributions of Tm, Mn, and O are presented in (**e–g**) respectively, as obtained by the EDS imaging with the SEM.

**Figure 4:**
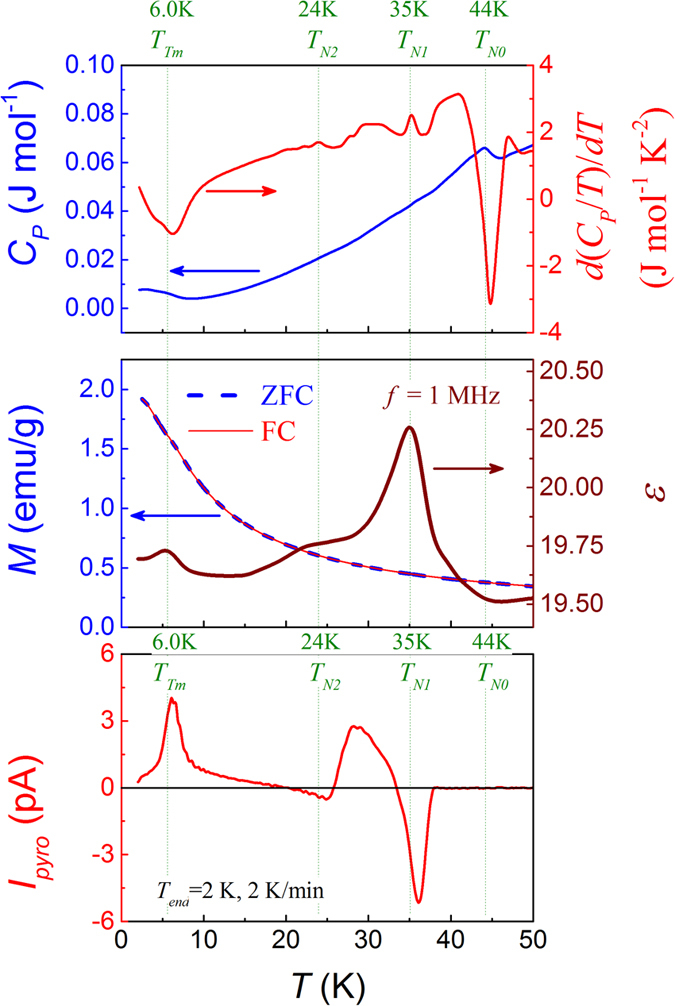
Measured specific heat *C*_*P*_ and its derivative *d*(*C*_*P*_/*T*)/*dT* (**a**) magnetization *M* under the FC and ZFC modes, and dielectric constant at *f* = 1 MHz (**b**) and pyroelectric current *I*_*pyro*_(*T*) at *T*_*end*_ = 2 K, *E*_*P*_ = 10 kV/cm, and a warming rate of 2 K/min (**c**) as a function of *T* respectively.

Before discussing the magnetically induced ferroelectricity data in details, we present one representative pyroelectric current *I*_*pyro*_(*T*) curve as shown in Fig. 4(c) measured at *T*_*end*_ = 2 K with a warming rate of 2 K/min. The data reliability will be identified later. First, one sharp valley at ~*T*_*N1*_ and one sharp peak at ~*T*_*Tm*_ are observed, consistent with the anomalies in the *ε*(*T*) curve. The sharp valley indicates the appearance of electric polarization at *T*_*N1*_. The peak at ~*T*_*Tm*_ indicates another FE transition and the generated electric polarization should be assigned as *P*_*LT*_ along the *a*-axis (shown in Fig. 1(b))^31^,^34^. Second, a broad peak between *T*_*N1*_ and *T*_*N2*_ is identified, suggesting the existence of third electric polarization which ensues gradually with decreasing *T*. Here, the most important feature is the negative current valley at ~*T*_*N1*_ and broad positive peak at *T*_*N2*_ < *T* < *T*_*N1*_, intimating the existence of two antiparallel polarizations, distinctly different from the reported data on single crystal samples^32^,^33^. This behavior is however similar to earlier data on polycrystalline DyMn_2_O_5_^29^,^39^. It may come to us immediately that the two polarizations are the components of a FIE state, giving rise to the polarization flop with decreasing *T* at certain temperature. Below *T*_*N2*_, the two polarizations compete with each other, leading to the flat grade between *T*_*N2*_ and *T*_*Tm*_. A detailed discussion on this FIE model will be performed later.

### Pyroelectric current and electric polarization

The issue of top priority here is the reliability of measured *I*_*pyro*_(*T*) data, and this issue is critical for a growing understanding of the underlying physics. The pyroelectric current method is sometime questioned since the measured “pyroelectric” current has been often questioned to include other current contributions such as trapped charges^29^,^30^,^42^. A careful clarification of these contributions if any should be made. We have performed the following measurements for this clarification.

**Figure 5:**
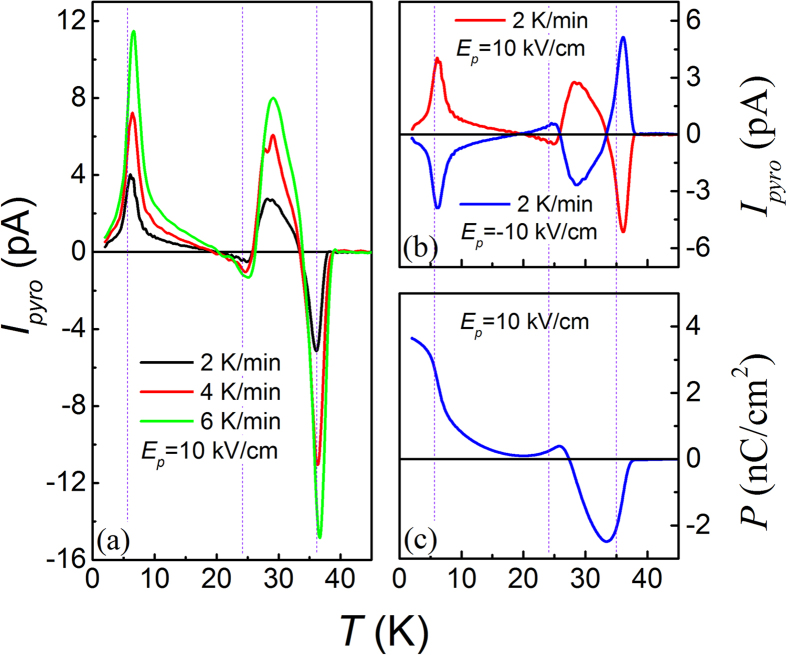
Measured *I*_*pyro*_(*T*) curves at three warming rates at *E*_*P*_ = 10 kV/cm (**a**) *I*_*pyro*_(*T*) curves at *E*_*P*_ = ±10 kV/cm and warming rate of 2 K/min (**b**) evaluated *P*(*T*) curve at *E*_*P*_ = 10 kV/cm (c). *T*_*end*_ = 2 K for all the cases.

First, we checked the *I*_*pyro*_(*T*) data at different warming rates given the fixed electric poling field *E*_*P*_ = 10 kV/cm and *T*_*end*_ = 2 K, as shown in Fig. 5(a). The three peaks/valleys against varying warming rate are non-shifted from one and another, and the under-curve area is roughly proportional to the warming rate, revealing that the measured current signals have no contribution from the de-trapped charges during the sample warming. Otherwise one will observe the shifting of these peaks/valleys towards the high-*T* side with increasing rate. Second, the nearly symmetric *I*_*pyro*_(*T*) curves with respect to axis *I*_*pyro*_ = 0 under *E*_*P*_ = ±10 kV/cm respectively, as plotted in Fig. 5(b), also evidence the reversible electric polarization. The as-evaluated *P*(*T*) curve is presented in Fig. 5(c), indicating clearly the appearance of a negative polarization around *T*_*N1*_, the polarization flop from negative value to positive one around *T*_*N2*_, and another FE transition around *T*_*Tm*_, respectively. This polarization flop is the character of a typical FIE system^29^,^38^.

**Figure 6:**
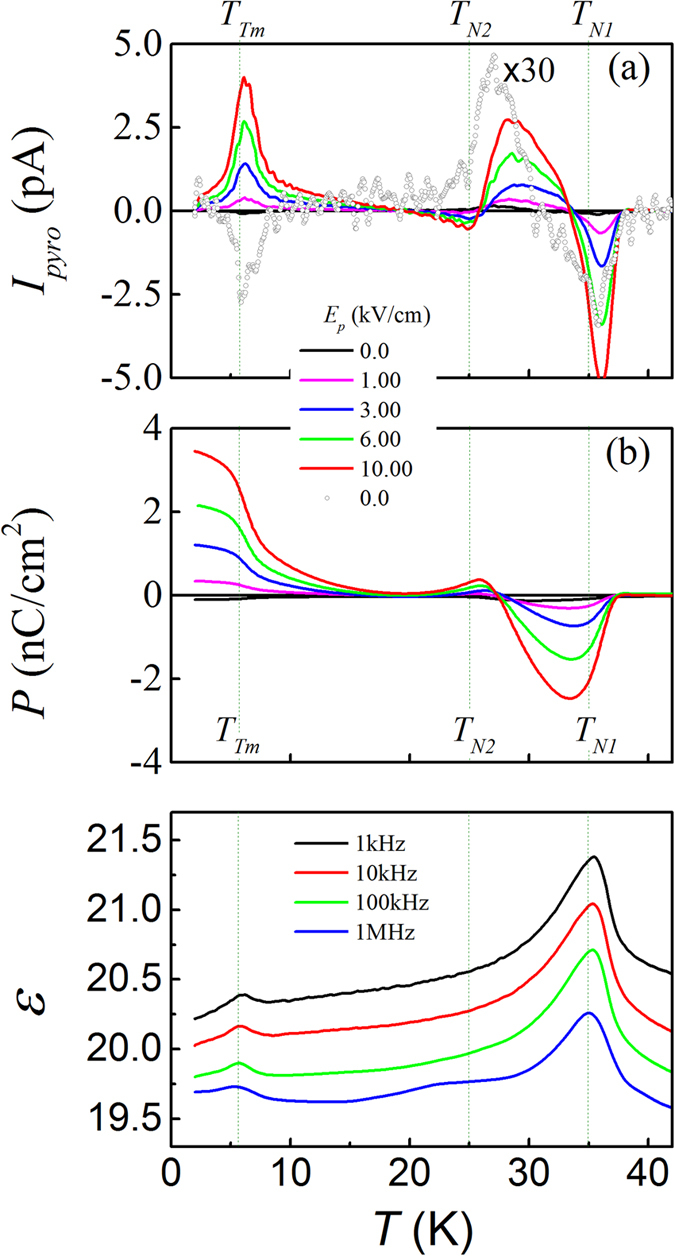
Measured *I*_*pyro*_(*T*) curves at different poling fields *E*_*P*_ as labeled (**a**). Evaluated *P*(*T*) curves from these *I*_*pyro*_(*T*) curves (**b**). *T*_*end*_ = 2 K for all the cases. Measured dielectric constant *ε*(*T*) curves at several different frequencies (**c**).

For further checking the pyroelectric origin for the *I*_*pyro*_(*T*) data, we plot the *I*_*pyro*_(*T*) curves measured at different *E*_*P*_ in Fig. 6(a). We first look at the data at *E*_*P*_ = 0 which are indeed very weak and can’t be identified unless the data are magnified for 30 times. The repeated measuring cycling shows the similar features: weak valley around *T*_*N1*_, weak bump right above *T*_*N2*_, and another weak valley or bump (history-dependent) around *T*_*Tm*_, respectively. Given the fact that the sample was not pre-poled electrically, these weak features suggest the existence of FE phase transitions around *T*_*N1*_, *T*_*N2*_, and *T*_*Tm*_ respectively. The electric pre-poling treatments under increasing *E*_*P*_ enable a set of *I*_*pyro*_(*T*) curves with non-shifted valleys/peaks but increasing valley/peak magnitudes. This fact also suggests no remarkable contribution from the de-trapped charges which otherwise would shift the valleys/peaks with increasing *E*_*P*_. The corresponding *P*(*T*) curves evaluated from the *I*_*pyro*_(*T*) data are plotted in Fig. 6(b), giving rise results consistent with the above discussion.

The measured *ε*(*T*) curves under an *ac*-field of 0.1 V/cm and different frequencies *f* are also plotted in Fig. 6(c) for checking the FE phase transitions. All these curves have the anomalies at *T*_*N1*_ and *T*_*Tm*_, while weak shoulders around *T*_*N2*_ appear only in the high-*f* curves. It is noted that the highest frequency used for the *ε*(*T*) measurements is 1.0 MHz which is sufficient to suppress any charge de-trapping under such a weak electric field. In the other word, these anomalies would be absent if the *I*_*pyro*_(*T*) signals are from the non-pyroelectric contributions. Furthermore, it is found that the dielectric peak at *T*_*N1*_ is quite broad, suggesting that the FE phase transition is not sharp. However, this phase transition would not be diffusive since no remarkable frequency dispersion can be identified^34^,^42^. The another possible reason is that the broad peak may come from a superposition of more than one FE phase transitions such as the consecutive appearance of the two electric polarizations of the FIE system, as to be proposed below. On the other hand, it is understandable that the polarization flop as proposed at *T*_*N2*_ may not necessarily produce significant dielectric response. Anyhow, to this end, one may trust that the measured *I*_*pyro*_(*T*) data are indeed contributed from the pyroelectric current and the FE phase transitions do appear at *T*_*N1*_ and *T*_*Tm*_, while a polarization flop at *T*_*N2*_ is argued.

### Path-dependent electric polarization

**Figure 7:**
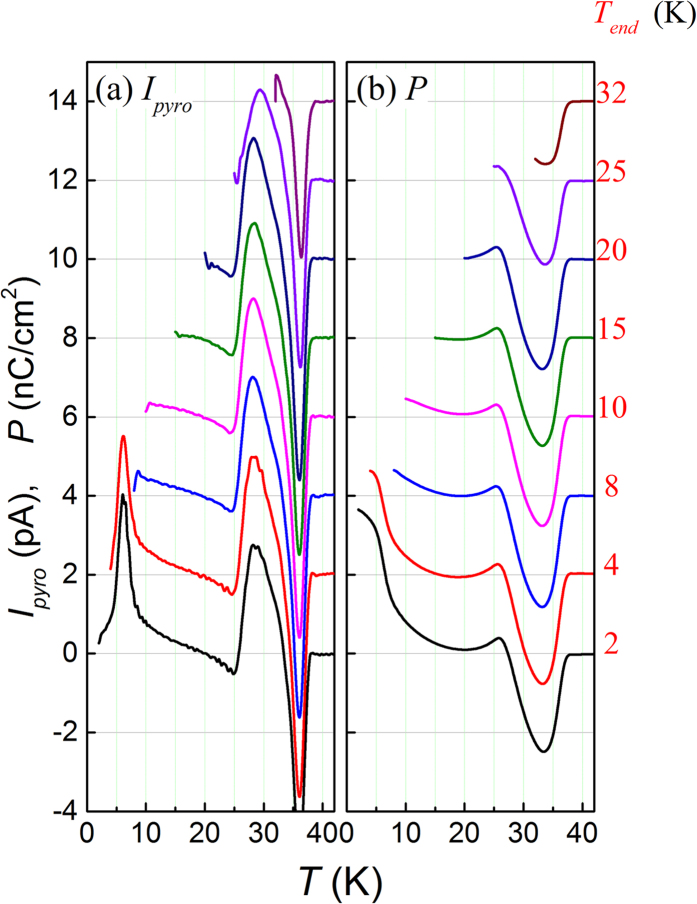
Measured *I*_*pyro*_(*T*) curves (**a**) and evaluated *P*(*T*) curves (**b**) for different values of *T*_*end*_ as marked. The warming rate is 2 K/min and *E*_*P*_ = 10 kV/cm. For data clarification, these curves are shifted from one and another vertically.

In order to understand the nature of the observed *P*(*T*) dependence, we have performed a set of *I*_*pyro*_(*T*) measurements given a fixed *E*_*P*_ = 10 kV/cm but different *T*_*end*_. The measured *I*_*pyro*_(*T*) curves and evaluated *P*(*T*) curves are plotted in Fig. 7(a,b) respectively where the curves are shifted vertically for clarification. Several critical features deserve for highlighting here. First, the major features of the *I*_*pyro*_(*T*) curves above *T*_*end*_ remain essentially unchanged, showing that the data are path-independent. In other words, this set of experiments demonstrates that the pyroelectric current is only *T*-dependent and has nothing to do with the measurement sequences (e.g. different *T*_*end*_). Second, the peaks around *T*_*Tm*_ and *T*_*N2*_ and the valley at *T*_*N1*_ are seemingly irrelevant with each other and the disappearance of one feature does not affect the others. Third, the polarization flop occurring around *T*_*N2*_ remains unaffected until *T*_*end*_ > *T*_*N2*_. What surprises us is that the negative polarization remains as *T* approaches *T*_*N1*_, noting that the sample is positively poled. This unusual behavior will also be discussed later.

## Discussion

**Figure 8:**
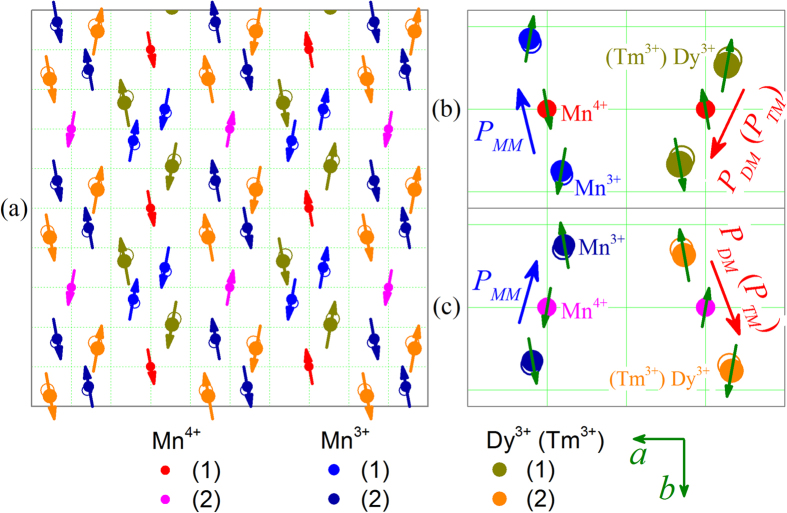
(**a**) The *ab*-plane projected ionic and spin configurations of DyMn_2_O_5_ and TmMn_2_O_5_ is assumed to have similar configurations, where the arrows indicate the spins, open circles mark the original ionic sites without inclusion of the magnetic interactions, and solid circles the final ionic sites with the magnetic interactions. (**b,c**) The four blocks each of which consists of three neighboring magnetic cation ions. The symmetric exchange striction in each block leads to shifting of the ions, generating a local electric polarization as indicated by the long arrow, and the as-generated polarizations *P*_*MM*_ and *P*_*DM*_ (*P*_*TM*_) are roughly antiparallel, forming a ferrielectric lattice.

We would like to address that all these features support the argument that the observed *P(T)* dependence is the consequence of a FIE system. Before we discuss this FIE scenario, it is noted that the *I*_*pyro*_*(T)* peak around *T*_*Tm*_ is contributed from the LCM phase generated polarization *P*_*LT*_ aligned along the *a*-axis^31^,^34^,^35^. Due to the polycrystalline nature of the samples, its contribution can be detected here. Similar to the FIE model in DyMn_2_O_5_, see Fig. 8, Fig. 8(a) shows the ionic and spin configurations on the ab-plane of DyMn_2_O_5_, which consist of ordered Mn^4+^, Mn^3+^, and Dy^3+^ occupations in the lattice. Clearly, the ab-plane spin configuration can be divided into four sub-groups as shown in Fig. 8(b,c)^39^. If the weak noncollinear components of these spins are not considered, these sub-groups actually are the ↑↑↓ or ↓↓↑ blocks. Each of these blocks contributes one local electric dipole due to the ionic displacement, consulting to the symmetric exchange striction for ferroelectricity generation. Immediately, one recalls that the ab-plane constitutes a FIE lattice of two roughly antiparallel polarization components: One component can be generated by the symmetric exchange striction in the Mn^3+^-Mn^4+^-Mn^3+^ blocks, denoted as *P*_*MM*_, and the other can be generated by the symmetric exchange striction in the Tm^3+^-Mn^4+^-Tm^3+^ blocks, denoted as *P*_*TM*_ which is antiparallel to *P*_*MM*_. Here, we applies it to the present case of TmMn_2_O_5_ for explaining the observed *P(T)* behaviors. The only revision to Fig. 8(a~c) is to replace the Dy^3+^ ions with the Tm^3+^ ions. In principle, it is believed that the strong Tm-Mn coupling enables the gradual ordering of Tm^3+^ spins in coherence with the Mn^3+^/Mn^4+^ spin ordering below *T*_*N1*_. The independent Tm^3+^ ordering occurs at *T*_*Tm*_ due to the competition of the Tm-Tm exchange over the Tm-Mn coupling, contributing to the LCM phase around *T*_*Tm*_.

The proposed physical sequence upon decreasing *T* is as the following. As *T* falls down to *T*_*N1*_, the Mn spins begin to order into the CM phase which generates polarization *P*_*MM*_. In spite of the claimed CM-ICM phase transition at *T*_*N2*_, no sufficient evidence with the absence of ferroelectricity in the CM phase is available. Therefore, it can be argued that this CM-ICM phase transition does not affect the *P*_*MM*_ very much. Since the Mn-Mn exchange is strong, suggesting that the spin ordering at *T*_*N1*_ is sharp and the FE phase transition finishes in a narrow T-range. On the other hand, the Tm-Mn coupling enables the coherent Tm^3+^ spin ordering with the Mn spin ordering, leading to polarization *P*_*TM*_ which is roughly antiparallel to *P*_*MM*_. This phase transition would be relatively diffusive since the Tm^3+^ spin ordering is induced by the Tm-Mn coupling as the second-order exchange. Consequently, the total polarization *P = P*_*MM*_ + *P*_*TM*_ as a function of *T* can be complex and a polarization flop event may occur.

**Figure 9:**
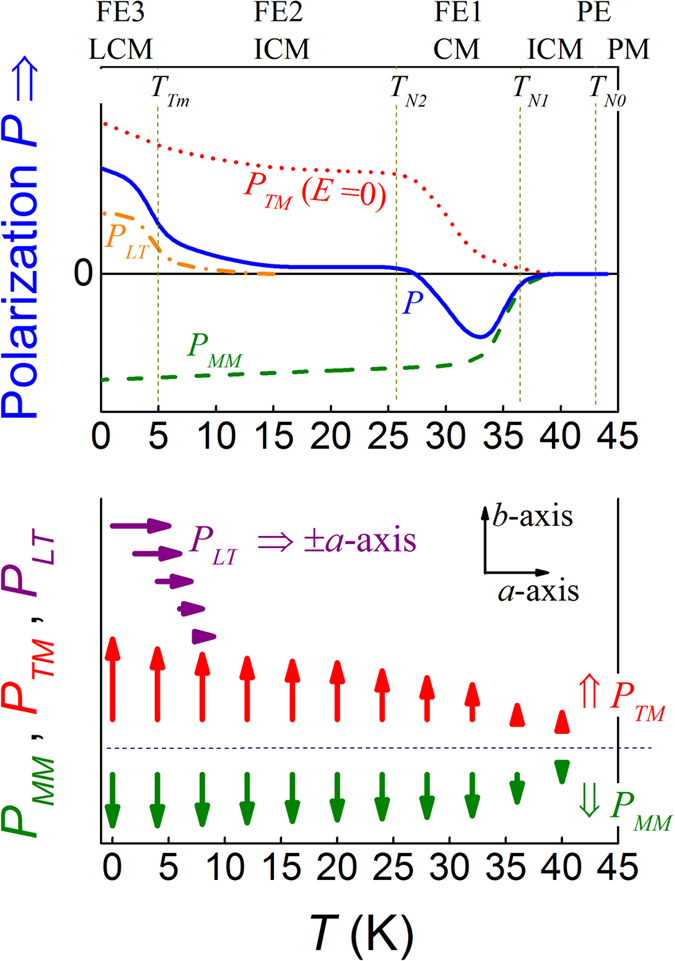
(**a**) Measured *P*(*T*) curve at the warming rate of 2 K/min and *E*_*P*_ = 10 K/min, and the assumed polarization components *P*_*MM*_(*T*), *P*_*TM*_(*T*), and *P*_*LT*_(*T*), as a function of *T* respectively. (**b**) A schematic drawings of the three polarization components (arrows in colors) at different *T*.

The observed *P(T)* curve suggests that inequality |*P*_*TM*_| > |*P*_*MM*_| should be satisfied below *T*_*N1*_. The proposed two components as a function of T in a qualitative sense, are plotted in Fig. 9(a). The two FE sublattices coherently constitute the FIE lattice and the two polarization components as a function of *T* respectively are schematically drawn just for a guide of eyes. When the low-*T* polarization component *P*_*LT*_ is summed to the total *P* = *P*_*MM*_ + *P*_*TM*_ + *P*_*LT*_, the observed *P(T)* curve (blue) can be nicely reproduced, as shown in Fig. 9(a) too. The three polarization components at different *T* can be schematically mapped by arrows in Fig. 9(b).

It has been repeatedly confirmed that the Mn spin orders in RMn_2_O_5_ systems are highly robust against external magnetic field^38^,^39^,^40^. A magnetic field of several Tesla seems not to shake much the Mn spin orders. Different from this property, one is aware of the much soft Tm^3+^ spin orders against magnetic field, due to the fact that the 4*f*-4*f* exchange is weak with respect to the Mn-Mn exchange^43^,^44^. The Tm-Mn coupling is much stronger than the 4*f*-4*f* exchange although it is relatively weaker than the Mn-Mn exchange. Therefore, both *P*_*TM*_ and *P*_*MM*_ are robust against H but *P*_*MM*_ is the highest robust. This difference allows an opportunity to check this FIE model by measuring the magnetoelectric response of TmMn_2_O_5_. Given a fixed *T*, applying a magnetic field certainly destabilizes the Tm^3+^ spin orders and thus completely suppresses polarization component *P*_*LT*_, while component *P*_*TM*_ may be also partially suppressed but *P*_*MM*_ remain less affected.

**Figure 10:**
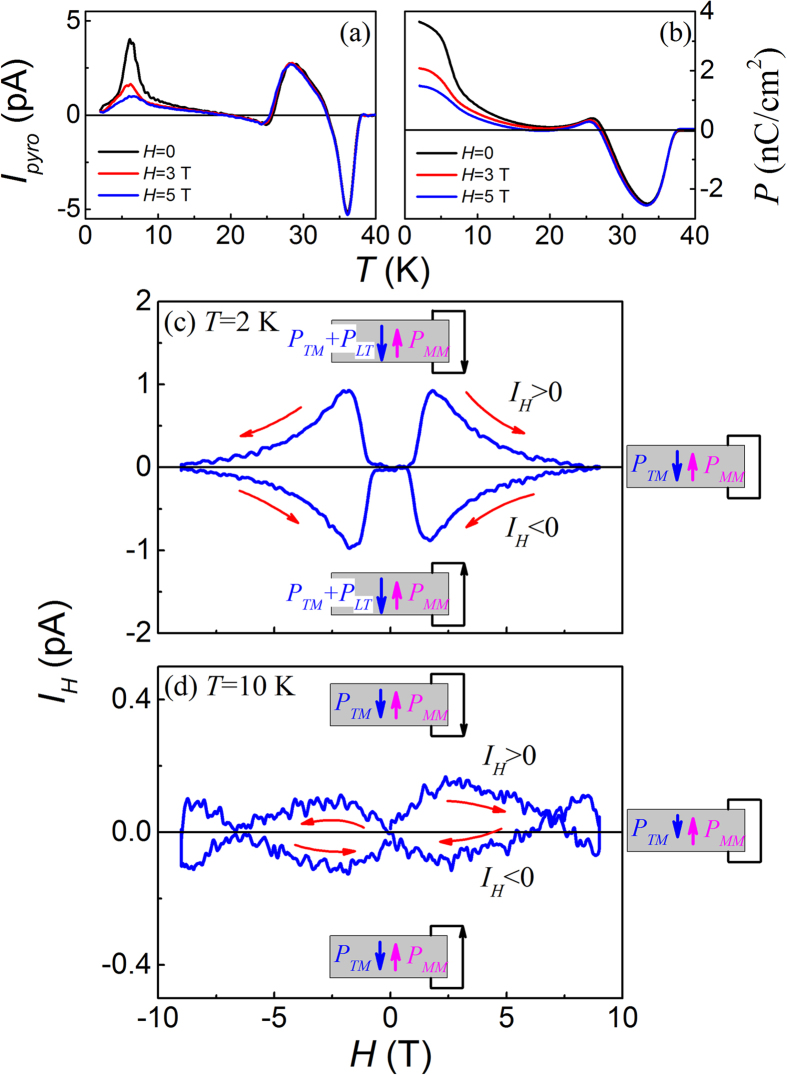
Measured *I*_*pyro*_(*T*) curves (**a**) and evaluated *P*(*T*) curves (**b**) under different magnetic fields *H* as labeled, with *T*_*end*_ = 2 K and *E*_*P*_ = 10 kV/cm. The iso-thermal *I*_*pyro*_(*H*) hysteresis loops at *T* = 2 K and 10 K are plotted in (**c,d**) respectively. The polarization components at various stages are drawn for a guide of eyes.

The above discussion is confirmed by the measured data, as shown in Fig. 10(a~d) respectively. On one hand, the *I*_*pyro*_*(T)* peak and the *P*_*LT*_ component in the *P(T)* curves around *T*_*Tm*_ are indeed suppressed by a field of ~5.0 T, while *P*_*MM*_ and *P*_*TM*_ remain less affected, as shown in Fig. 10(a,b). On the other hand, Fig. 10(c,d) show the magnetically induced current *(I*_*H*_) loops in response to the *H*-cycling at *T* = 2 K and 10 K. The *I*_*H*_ ~ *H* loop at 2 K clearly indicates the suppression of *P*_*LT*_ by increasing and decreasing *H* cycle. This feature becomes seriously weakened at *T* = 10 K where the weak *I*_*H*_ response is from the partially *H*-suppressed *P*_*TM*_, consistent with the above discussed FIE scenario too.

It should be noted that our proposed FIE model seems to reasonably explain our experimental results. However, the ceramic samples exhibit some intrinsic defects such as grain boundaries and voids, which have an influence on the polarization properties, although the use of polycrystalline may avoid the impact of self-polarized effect on polarization behaviors in our samples. Therefore, we felt that more research by testing of single crystal samples is needed to further support our claims.

Furthermore, an unsolved and puzzling issue regarding this FIE model is the appearance of negative *P* right below *T*_*N1*_ for both DyMn_2_O_5_ discussed earlier and TmMn_2_O_5_ here. In fact, the appearance of negative *P* right below *T*_*N1*_ is understandable if *T*_*end*_* *≪ *T*_*N1*_, because of |*P*_*TM*_| > |*P*_*MM*_| at *T *≪ *T*_*N1*_ and *E*_*P*_ > 0. However, as shown in Fig. 7, for *T*_*end*_ ~ 32 K at which |*P*_*TM*_| < |*P*_*MM*_| and thus the positive *E*_*P*_ would drive a positive *P* instead of negative *P* under a positive poling field. An understanding of this unusual feature is detrimental to the FIE model, which otherwise could be totally wrong.

So far, almost all of the literature discussing the multiferroicity of RMn_2_O_5_ has concentrated on the magnetically induced ferroelectricity below *T*_*N1*_ (*T*_*N0*_)^18^,^19^,^20^,^21^,^22^,^23^,^24^,^25^,^26^,^27^. This fact leaves a somehow misleading impression that RMn_2_O_5_ would be paraelectric above *T*_*N1*_. The only exceptional experiment was from V. Baledent *et al*. who demonstrated that RMn_2_O_5_ is actually a family of room ferroelectricity whose microscopic origin has nothing to do with magnetism, and it was argued that this room temperature ferroelectricity is structurally driven^45^. The magnetically induced FE polarization is simply an additional component to the room ferroelectricity. Unfortunately, due to the extremely large conductivity of single crystal RMn_2_O_5_ at *T* > 150 K, the FE polarization at high *T* can’t be measured. In the present work, our polycrystalline samples show better insulating property than single crystals, allowing a relatively sufficient electric poling at *T* up to ~300 K by a field of ~8 kV/cm, although the electrical insulativity of the samples are still far from sufficient to obtain reliable *P-E* hysteresis. The high-*T* FE phase transition temperature has not yet been determined.

**Figure 11:**
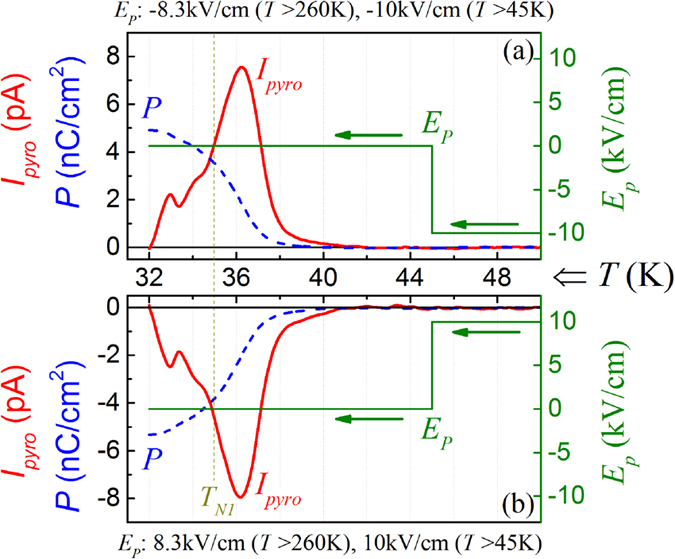
Measured *I*_*pyro*_(*T*) curve and evaluated *P*(*T*) curve for two specific cases. The sample was cooled down from room temperature with an electric poling down to 45 K, and then further down to 32 K with zero electric bias, followed by warming at a rate of 2 K/min during *I*_*pyro*_(*T*) data are is probed: (**a**) *E*_*P*_ < 0 and (**b**) *E*_*P*_ > 0. The poling sequences for the two cases are marked.

Nevertheless, the claimed room ferroelectricity for TmMn_2_O_5_ allows us an opportunity to explore the origin for the negative *P* right below *T*_*N1*_. We perform the following experiments: an electric field *E*_*P*_(*T*), which is gradually increased with decreasing *T*, is imposed to the sample during the sample cooling. The value of *E*_*P*_ is increased to 10 kV/cm (or −10 kV/cm) at 260 K down to *T* ~ 45 K which is slightly higher than *T*_*N0*_ = 44 K. Then the poling field is removed and the sample is further cooled down to 32 K which is below *T*_*N1*_. Subsequently, the sample is sufficiently short-circuited electrically and then the sample is warmed at a rate of 2 K/min up to 50 K, during which the *I*_*pyro*_(*T*) is measured. The measured data are presented in Fig. 11(a,b) respectively, where the *E*_*P*_(*T*) data are also inserted for reference.

Surprisingly, one observes the positive *I*_*pyro*_ peak (positive *P*) around *T*_*N1*_ when *E*_*P*_ < 0 above *T*_*N0*_ (Fig. 11(a)), and the negative *I*_*pyro*_ peak (negative *P*) around *T*_*N1*_ when *E*_*P*_ > 0 above *T*_*N0*_ (Fig. 11(b)), noting that no electrical bias is imposed on the sample between *T*_*N0*_ and *T* = 32 K. This implies that the negative *P* valley just below *T*_*N1*_ in our whole package of measurements is induced by the electric poling process far above *T*_*N0*_. This is the reason for the observed negative *P* valley in Fig. 7 at *T*_*end*_ = 32 K and below.

Assuming that TmMn_2_O_5_ is already a ferroelectric at room temperature and its polarization is *P*_*0*_, which is positive if *E*_*P*_ > 0 and negative if *E*_*P*_ < 0. This structurally induced *P*_*0*_ may align the magnetically induced polarization *P*_*MM*_ in opposite direction via e.g. the ferroelastic effect or so far unknown magneto-lattice coupling mechanism^46^,^47^. In the other words, *P*_*MM*_ is always antiparallel to *P*_*0*_ which is however aligned by the electric poling. Although such a coupling or ferroelastic effect has not yet been understood and will be further investigated, all of the observed phenomena in our experiments can be well explained, given this *P*_*MM*_↓↑*P*_*0*_ assumption.

## Conclusion

In conclusion, we have performed extensive measurements of the magnetically induced ferroelectricity of TmMn_2_O_5_ in polycrystalline form, focusing on the ferrielectric nature and its magnetic origins. It is revealed that the ferroelectric polarization contains several components which appear respectively in various temperature ranges and is suggested to be generated by different microscopic mechanisms. These components include the polarization *P*_*MM*_ generated by the Mn^3+^-Mn^4+^-Mn^3+^ collinear spin block via the symmetric exchange striction, the polarization *P*_*TM*_ generated by the Tm^3+^-Mn^4+^-Tm^3+^ collinear spin block via the symmetric exchange striction, and the polarization *P*_*LT*_ generated by long-range noncollinear Tm^3+^ spin ordering via the asymmetric exchange striction. The Tm^3+^-Mn^4+^-Tm^3+^ collinear spin ordering is driven by the strong Mn-Tm coupling. The two components *P*_*MM*_ and *P*_*TM*_ are antiparallel, constituting two ferroelectric sublattices of a ferrielectric system. The polarization flop with decreasing temperature is observed as a representative character of the ferrielectricity. It is suggested that this magnetically induced ferrielectricity of TmMn_2_O_5_ is likely an additional ingredient to the high-temperature ferroelectricity which is structurally driven. The present work represents a growing understanding of the complicated multiferroic behaviors in RMn_2_O_5_ compounds.

## Methods

In our experiments, polycrystalline TmMn_2_O_5_ samples were prepared by the standard solid-state reaction method. Stoichiometric amounts of Tm_2_O_3_ (99.99%) and Mn_2_O_3_ (99%) powder were thoroughly ground and then fired at 980 °C for 24 h in a flowing oxygen atmosphere. The resultant powder was re-ground and granulated using 4 wt% poly vinyl alcohol (PVA) solution and then pelletized under a pressure of 9 MPa into disks of 11.5 mm in diameter. The disk samples were sintered at 1080 °C for 48 h in a flowing oxygen atmosphere in prior to natural cooling down to room temperature. The as-prepared samples were submitted to a set of microstructural characterizations. The crystallinity and lattice structure were checked using the X-ray diffraction (XRD) (PANalytical X’Pert PRO diffractometer) with the Cu-*K*_*α*_ radiation at room temperature. The data were refined using the Rietveld method. The scanning electron microscopy (SEM, Ultra 5, Zeiss) and the associated EDS mapping were used to check the grain morphology and chemical distribution.

The isometric specific heat (*C*_*P*_) of the sample as a function of *T* was measured in the standard procedure using a physical properties measurement system (PPMS, Quantum Design Inc.) installed inside a well-shielded space to insure extremely low electrical and thermal noise (background). The electrical noise can be as low as 0.02 pA as probed by the Keithley 6430 electrometer. The evaluated *d*(*C*_*P*_/*T*)/*dT* ~ *T* data are used to mark these phase transition points. The *dc* magnetization *M*(*T*) data at the field-cooling (FC) and zero-field-cooled (ZFC) modes were obtained using the vibrating sample magnetometer (VSM) integrated with the PPMS system. The cooling and measuring fields were both 100 Oe, sufficiently low so that the magnetic field driven side-effects if any are as weak as possible.

For the electrical measurements, disk-like samples of 11.5 mm in diameter and 0.17 mm in thickness were deposited with Au electrodes on each side. The dielectric constant *ε* as a function of *T* at various frequencies (*f*) covering six orders of magnitude was measured using the E4980A precision LCR meter. The electric polarization *P* as a function of *T* was evaluated from the pyroelectric current *I*_*pyro*_-*T* data. The measurement was carried out following the procedure below. First, the sample in the plate capacitor geometry was cooled down to 150 K without electrical bias and then an electric poling field *E*_*P*_ = ± 10 kV/cm was applied to the sample during further cooling at a rate of 2 K/min down to a given end temperature *T*_*end*_. Then the sample capacitor was electrically short-circuited for sufficient time at *T*_*end*_, followed by a slow sample heating until a temperature higher than *T*_*N0*_, during which the electric current (*I*_*tot*_) released from the capacitor was recorded using the Keithley 6430 electrometer.

A set of additional experiments were performed to check whether the probed current *I*_*tot*_ from the capacitor is solely from the pyroelectric current *I*_*pyro*_ or not. These experiments include the measurement of dependence *ε*(*f*, *T*), released current curves *I*_*tot*_(*T*) at several heating rates, and isothermal magneto-current *I*_*H*_ in the *H*-cycling at a rate of 150 Oe/s. The *I*_*pyro*_(*T*) data under various magnetic fields *H* were also collected. The *T*_*end*_ was varied from 2 K to *T* < *T*_*N1*_. Furthermore, the polarization current method was also employed to qualitatively the sample’s ferroelectricity. The current passing through the sample under an electric field of 10 kV/cm, which should contain both the leaky current (*I*_*E*_) and polarization current (*I*_*P*_), was measured too. Assuming that *I*_*E*_(*T*) decreases monotonously with decreasing *T*, the *I*_*P*_ can be extracted although it may not be quantitatively accurate. Unfortunately, for TmMn_2_O_5_, the current passing across the samples was too big to be possible for extracting the polarization current of tens of pA, suggesting the incapability of this method in the present case.

## Additional Information

**How to cite this article**: Yang, L. *et al*. Understanding the multiferroicity in TmMn_2_O_5_ by a magnetically induced ferrielectric model. *Sci. Rep*. **6**, 34767; doi: 10.1038/srep34767 (2016).
